# First steps in co‐designing an online patient decision support tool for enhanced medication management in older people

**DOI:** 10.1111/ajag.70053

**Published:** 2025-06-13

**Authors:** Temitope Esther Afolabi, Sarah N. Hilmer, Christopher Etherton‐Beer, Lisa Kouladjian O'Donnell

**Affiliations:** ^1^ Laboratory of Ageing and Pharmacology, Faculty of Medicine and Health, Kolling Institute The University of Sydney and the Northern Sydney Local Health District St Leonards New South Wales Australia; ^2^ School of Medicine University of Western Australia Crawley WA Australia; ^3^ Faculty of Medicine and Health, School of Pharmacy The University of Sydney Camperdown New South Wales Australia

**Keywords:** ageing, co‐design, health technology, patient decision support, steering committee

## Abstract

**Objective:**

To develop a preliminary version (minimum viable product [MVP]) of an online medication management patient decision support for older people informed by a stakeholder committee (SC).

**Methods:**

A SC comprising of consumers, health‐care practitioners and researchers was recruited to inform the development of the MVP. The SC met via videoconferencing, individually or in groups, with investigators to provide guidance and advice on the design and features of the MVP. Brief content analysis was performed on data extracted from video‐recorded meetings, meeting minutes, notes and email correspondence to inform the MVP development.

**Results:**

The SC comprised two older people, one carer, pharmacist, nurse, geriatrician, general practitioner and two digital health researchers (*n* = 9) from three Australian states. In collaboration with the SC, an MVP called *My Medicine Goals* (mymedicinegoals.com) was developed for older people. It supports consumers to document and update their medicines list; identify, plan and communicate goals of care; and provides a medicines information resource hub. Other tools include interactive versions of the revised Patient's Attitudes Towards Deprescribing questionnaire and self‐assessment of medication risks.

**Conclusions:**

This project presents an approach empowering older people and carers to play active roles in co‐design research to develop relevant and impactful online patient‐facing health‐care applications for older people.

**Practice impact**

Partnership with a diverse stakeholder committee achieved collaborative development of a patient decision support for medication management by older people—a critical first step in the co‐design approach.

## 1 INTRODUCTION

Older people are very susceptible to harm from medication use, as they are the largest users of medications, coupled with age‐related physiological, pharmacokinetic and pharmacodynamic changes.^1^ As people age, they are more likely to have polypharmacy (≥5 medications), which is associated with increased risks of adverse drug reactions, falls, poor quality of life, high health‐care utilisation, significant morbidity and premature mortality.^2^ Much research has provided health‐care practitioners (HCPs) with evidence‐based strategies and tools to mitigate polypharmacy and optimise patient outcomes, including the development of a wide range of online clinical decision support (CDS) tools for HCPs.^3^, ^4^ Clinical decision support tools augment complex decision‐making processes by HCPs and facilitate shared decision‐making with consumers.^4^ Similarly, consumers (especially sub‐groups with polypharmacy, multimorbidity and older people) are faced with complex decisions about their health and may benefit from the development of comparable support systems.^5^

The extensive research on CDS for HCPs has inspired the advent of patient decision support (PDS) tools such as Ottawa Personal Decision Guide,^6^ My Care Companion Decision Aid,^7^ online patient portals and a range of other patient‐facing software innovations.^5^ Patient decision support tools have improved patient knowledge of treatment options, involvement in one's care and satisfaction with decisions, and positively impacted health outcomes.^5^, ^6^, ^7^ Support for online PDS continues to mount in today's evolving technological era.^4^, ^5^ Emerging evidence indicates that use of online PDS tools by patients and their carers is high, including among older people.^8^

In this study, we aimed to co‐design an online PDS for older people to assist with medication management. This required an understanding of the technological requirements of older people, and how to design technologies that meet these needs. Involving older people in co‐design can improve knowledge transfer and co‐create technological solutions for older people.^9^ Co‐design is an emerging field in health research and is broadly defined as the process of engaging consumers as equal partners in research.^10^ Benefits of effectively employing co‐design approaches include development of a product or service of increased relevance, greater stakeholder uptake and reduced research waste.^11^ There is no single ideal approach to co‐design owing to a lack of standardised terminology and conceptual definitions.^11^, ^12^ Greenhalgh et al.^11^ suggest that researchers should tailor their co‐design approaches to their individual contexts. In keeping with this notion and to provide structure, we have adapted Sanders and Stappers'^13^ co‐design framework to our context to develop an online PDS for older people. The significance of this study lies in its contribution to the understanding of co‐designing online medication management tools with older people. This paper presents first steps in co‐designing an online medication management PDS for older people and features of the preliminary PDS. This work is part of a larger study, the Frailty Interventions Through Sex‐Specific Therapies (FITTEST) study clinical trial (ACTRN12624000693527), supporting the management of frail older people. Our PDS forms one of the four components of the FITTEST study.

## 2 METHODS

**FIGURE 1:**
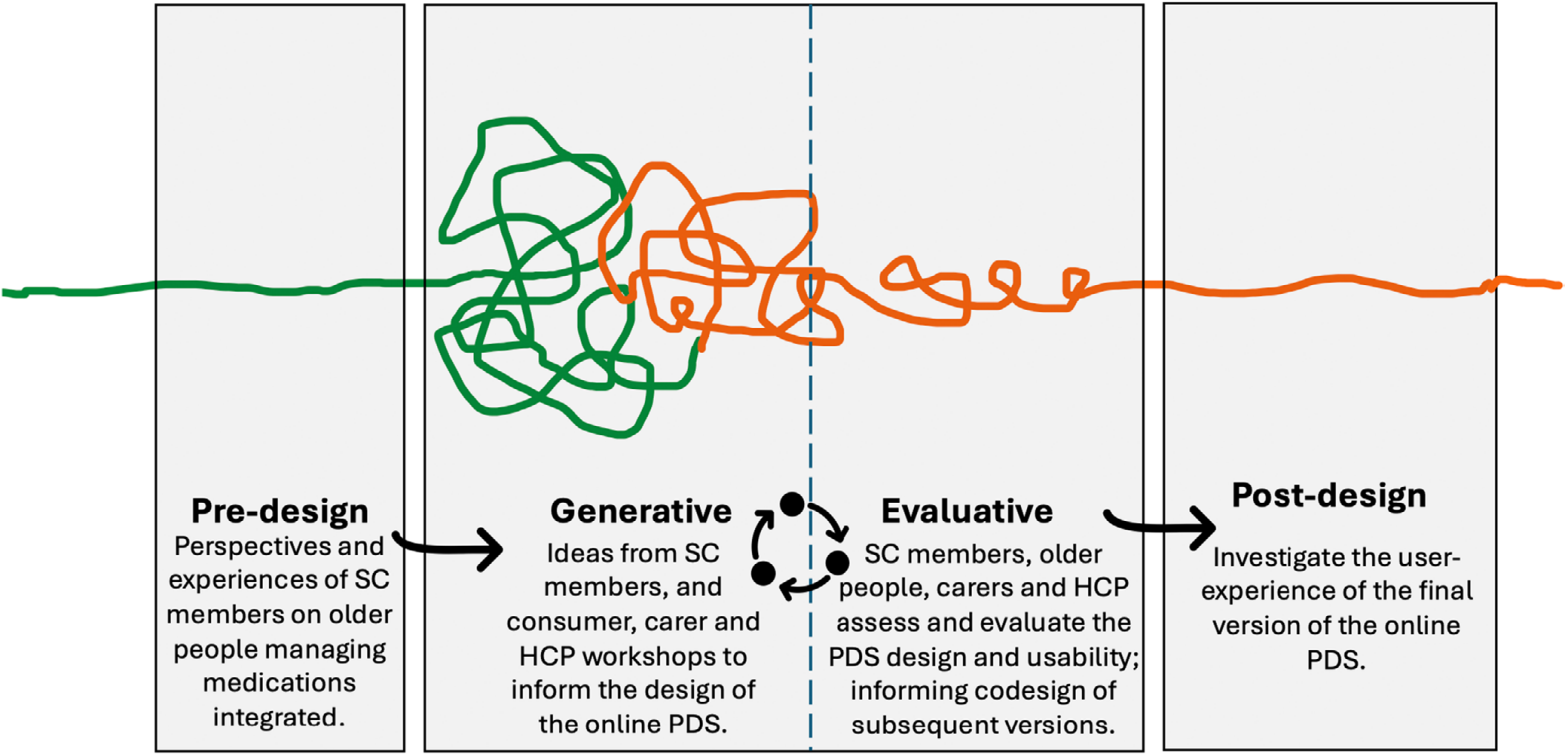
Sanders and Stappers^13^ codesign framework adapted to the development of an online Patient Decision Support (PDS) for medication management by older people. Green lines represent completed parts of the project, orange lines represent parts of the project yet to be completed, and the broken line between generative and evaluative depicts the iterative process between these two phases. HCP, health‐care practitioner; PDS, patient decision support; SC, Stakeholder Committee.

To co‐design an online PDS website for older people to assist with medication management, we applied Sanders and Stappers'^13^ co‐design framework, which describes the co‐design process as four interconnected phases: (a) Phase 1: Predesign; (b) Phase 2: Generative; (c) Phase 3: Evaluative; and (d) Phase 4: Postdesign. This paper describes co‐design Phases 1 and 2, with subsequent Phases (3 and 4) to be covered in future publications. Noorbergen et al.,^14^ who contextualised this co‐design framework and constructed guidelines for its application to mobile health systems development, described the predesign phase as centred around understanding the context and experiences of the intended users of mobile health systems. Applying this approach to our context (Figure 1), in Phase 1, we set out to understand the experiences, context and goals of our intended users, that is older people and carers using an online PDS, by recruiting a stakeholder committee (SC) comprised of older people, informal carers, HCPs and digital health researchers to govern the co‐design work and inform the development of a preliminary version of a PDS website.

The production of ideas, insights and concepts obtained from engaging a diverse SC, comprised the beginnings of the generative phase of our co‐design (Phase 2). In co‐design, the generative phase makes up a significant portion of the design process. This involves the co‐creation of conceptual artefacts such as mock‐ups (i.e. visual reproduction of the end‐product) and a minimum viable product (MVP). As presented by Mrklas et al.,^15^ an MVP (i.e. preliminary product) facilitates early testing of activities and allows for revising and refining ideas and concepts that may then be further designed and developed.^13^, ^14^, ^15^ In this paper, the preliminary version of the online PDS may be referred to as the MVP. In the generative phase of co‐design, the vision is tortuous, and iterative changes are anticipated as one moves back and forth between the generative and evaluative phases.^14^

### 2.1 Stakeholder Committee member selection

The Northern Sydney Local Health District Human Research Ethics Committee waived the need for ethics approval as this project involved consultations with a SC, in an advisory capacity. A SC was recruited to provide guidance and oversight. To recruit consumer members, an expression of interest (EOI) was advertised via email to members of the Australian Frailty Network (AFN) Consumer Network (*n* = 33 at time of recruitment) comprising older people and informal carers, seeking two to three individuals. The AFN consumer group was purposively selected to facilitate the recruitment of older people and informal carers who are actively involved in health research and advocacy for older people.

Eligibility criteria for SC consumer members included individuals aged 65 years and older taking regular medications (or is an informal carer for such a person) with interests in improving medication management for older people, good communication skills, access to the internet and the ability to actively participate in SC via videoconferencing. All EOIs received were reviewed by the investigators, and shortlisted consumers were invited to join the SC. The remaining SC members were recruited purposively via direct invitation to HCPs and researchers with known expertise relevant to the project. The following were key considerations in the selection of our SC: individual members' expertise, equity and diversity, budget for remuneration of committee members and efficiency of engagement with SC. The SC was capped at nine members, the upper limit recommended in co‐design guidelines, to capture a broader range of perspectives and expertise.^16^

### 2.2 Stakeholder Committee engagement

In accordance with the Six‐Step Stakeholder Engagement Framework,^17^ a term‐of‐reference was developed detailing the purpose of engagement, SC responsibilities, number of meetings, mode of involvement, duration of engagement and remuneration. All SC members returned their signed terms of reference to the investigators, providing their informed consent prior to inclusion in the study.

Interactions with the SC were via email and videoconferencing, which allowed for a diverse SC, with members originating from three Australian states (New South Wales, South Australia and Queensland). Each SC meeting ran for up to 60 min, including ‘check‐ins’ and Zoom breakout room discussions. A ‘check‐in’, as applied by McKellar et al.,^18^ is an opportunity for each member to introduce themselves and share whatever may be top of mind. A total of five SC meetings were conducted, with additional meetings anticipated across the remaining co‐design phases. Refer to Appendix S1 for a copy of the discussion questions that guided the SC meetings. A brief content analysis^19^ involving independent (TEA) and joint reviews (TEA, SH, LKO) of meeting recordings, minutes, notes and emails from SC members was conducted by the investigators to reach consensus on main discussion topics informing the MVP development. Table 1 below summarises our engagement with the SC.

**TABLE 1:** Timeline of events for engaging a project stakeholder committee (SC) in the development of a Minimum Viable Product (MVP) Patient Decision Support.

Recruitment of SC (September–October 2023) The Northern Sydney Local Health District Human Research Ethics Committee was consulted, and ethics approval deemed not required for involvement of a project SC. Recruitment of consumer members was conducted via an EOI process and remaining members were recruited purposively.
Overview of SC meetings (October–November 2023) All meetings ran for approximately 60mins and some meetings were repeated accommodating to the different time zones and member availabilities. The SC met individually or in groups, with investigators. The older SC members (C1 and C2) preferred meeting in the morning to fit around other commitments such as medical appointments. Meeting minutes were circulated to the SC and investigators.
Predesign phase Meetings 1 and 2: Introductions, project overview and initial discussions of MVP concept and design (held on two separate dates and times to accommodate the availabilities of all SC members). Videoconferencing breakout rooms: SC divided into two groups for breakout room sessions. Each group comprised of at least one consumer SC member, one digital health researcher SC member and a mix of other SC members and led by the investigators (TEA or LKO). This composition allowed all members to voice their opinions and share experiences freely, particularly for the older SC consumer members.
Generative design Meetings 3–5: Supplementary meetings reviewing key components of the MVP. SC members provided written feedback detailing their suggestions, ideas, and feedback, most of which were used to inform the design of the MVP.
Development of the MVP: *My Medicine Goals* website SC informed website design and build and agreed to the name *My Medicine Goals*. Preliminary website testings were conducted by SC members. My Medicine Goals is one of the interventions in the Frailty Interventions Through Sex‐Specific Therapies (FITTEST) study clinical trial (ACTRN12624000693527) supporting the management of frail older people.
Future SC meetings Planning subsequent phases of the project including completing the generative phase, commencing evaluation and postdesign work.

Abbreviations: EOI, expression of interest; FITTEST, Frailty Interventions Through Sex‐Specific Therapies; MVP, minimum viable product; SC, Stakeholder Committee.

## 3 RESULTS

### 3.1 Predesign Phase 1

Of the 33 consumers from the AFN Consumer Network, eight expressed their interest to participate in the SC. Based on the eligibility criteria, three consumers were shortlisted and subsequently accepted invitations to participate in the project SC. The SC comprised two older people (C1 and C2), one informal carer for older people (C3), one pharmacist (Ph1), one nurse (RN1), one geriatrician (G1), one general practitioner (GP1) and two digital health researchers (DHR1 and DHR2) who were independent of the research team (*n* = 9). The SC members included people with lived experience, people from culturally and linguistically diverse backgrounds, researchers and HCPs, lending the project rich insights that shaped the MVP's features, functionality and overall user experience.

Having SC members check‐in at the beginning of each meeting encouraged rapport building and open dialogue. Dividing the SC into smaller breakout room sessions of 3–4 people during meetings allowed for robust discussions and opportunities for all members to voice their opinions more freely. Limiting meeting lengths to 60 min was essential to reduce fatigue and maintain engagement considering the older consumer members in the group and to accommodate busy HCPs. All members participated during and outside SC meetings, evidenced by the robust discussions in meetings and emails received outside meetings. Members Ph1, DHR1, DHR2, C1 and C2 each provided comprehensive feedback via email, reviewed the MVP concept, design, content and usability. All SC consumer members C1, C2 and C3 described their experiences on the project SC as positive and have expressed their continued interest in participating on the SC. Data informing the MVP design were obtained from SC meeting recordings, meeting minutes and written feedback from SC members.

### 3.2 Generative Phase 2

My Medicine Goals (MMG) website, a preliminary online PDS assisting older people with medicines management, has been developed in collaboration with our SC. This website (mymedicinegoals.com) contains a dashboard for older people and informal carers to document and update their medicines list electronically via the website's Medicines List builder; identify and communicate Specific, Measurable, Achievable, Relevant, Time‐bound (SMART) health and medicine related goals with HCPs; and a medicines information hub. Other tools within MMG include interactive versions of the NPS MedicineWise Medicines Risk Screen, and the revised Patient's Attitudes towards Deprescribing (rPATD) questionnaire.^20^ The decision to include SMART goals and the rPATD questionnaire in MMG was informed by the clinician decision support tool, Goal‐directed Medication review Electronic Decision Support System.^21^ The Medicines List builder, an online tool for creating a personalised medicines list, was derived from the NPS MedicineWise medicines list. Incorporation of the other tools into MMG was informed by an unpublished scoping review of the grey literature. All tools were presented to the SC and subsequently refined.

After content analysis of the data, three main issues were identified: (a) website usability across diverse populations; (b) optimising the user experience; and (c) priorities of SC consumer members versus HCP members (Table 2).

**TABLE 2:** A summary of identified issues and SC member suggestions for future research.

Identified topics and issues	Related SC member quotes
(a) Website usability across diverse populations People from culturally and linguistically diverse backgrounds Individuals with multimorbidity Carers and families Polypharmacy and people with high pill counts Older people with low digital literacy	‘Is the design culturally appropriate for Aboriginal and Torres Strait Islander people?’ RN1 ‘My parents speak Mandarin; can the website translate to another language?’ C2
	‘My wife has many chronic conditions and takes a lot of medications … I can ask her what she thinks about this [website]’ C3
	‘Many old people have either a family member or carer that looks after, assists them with their medications … it is important that the study also captures the needs of carers and … is reflected on the website as well’ C3
	‘Many old people take lots of medicine, an online list can help them keep track’ C1
	‘Older people not as tech savvy as younger people’ C3
(b) Optimise the user experience Simplicity and enhancing the user experience Overall design and aesthetics Images used: cartoon versus real people	‘Older people not as tech savvy as younger people’ C3 ‘Keep it simple, easy flow’ C2 ‘Fewer clicks when navigating through the website’ C3 ‘Font could be bigger’ Ph1 ‘Enlarge font size and keep casing consistent’ DHR1 ‘Images should be consistent with the text’ C2 ‘Medicine names should appear as the active ingredient because that is what doctors write on scripts now’ C1 ‘I prefer brand names’ C2 ‘ … can an educational video explaining brand names vs generic names be developed?’ DHR1
	‘I like the colours, soft colours and the theme … looks great!’ C1 ‘The website colour scheme and design is great’ Ph1 ‘I really like the big buttons and simple questions and options’ DHR2 ‘Clean and professional looking’ C2 ‘Overall good amount of detail’ C1
	‘Current images on portal are stereotypical and demonstrate being unwell’ Ph1 ‘I actually like the cartoon images … looks good to me’ C2 ‘Let consumers in your focus groups decide between cartoon images versus real people images or a combination of both.’ DHR1
(c) Priorities of SC consumer members versus SC HCP members SC HCP members were more concerned with ensuring accuracy of the medicines list prepared by users SC consumer members expressed their value for having an online medicines list functionality built into the website compared to a paper medicines list	‘It may be difficult for older people to manage their own medicines list with guaranteed accuracy – is the intended purpose of the list just for empowerment and information for the older person and their carers, or is it intended as a useable list for health practitioners involved in clinical decision making?’ Ph1
	‘Paper lists gets lost’ C2 ‘They [referring to written medication lists] are not always up to date’ C1 ‘ … may not be legible’ C3 ‘A medicine list like this is not good for emergency because no one else can see what the patient is taking’ C2
Other issues to consider Use consumer‐ focused language Limited accessibility to the online portal in emergency situations due to login restrictions	‘Use of the term ‘patient’ throughout the portal implies that a person is ‘sick’… for consumer‐focussed language consider referring to users as a ‘person’ rather than a ‘patient’ irrespective of whether they are in hospital or not.’ Ph1 ‘Not good for emergency … you must enter email and password to access … can't do this if emergency’ C2
SC suggestions for future directions	
Running co‐design focus groups/workshops with a range of stakeholders including older people, caregivers and HCPs to capture various perspectives	
Integration of My Medicine Goals with electronic health records	
Listing corresponding side effects and related medication information alongside all medications the user includes in their online medicines list	
Developing a mobile application and/or desktop cloud version of the website for increased accessibility	
Barcode scanning of medication boxes and prescriptions for improved accuracy of the information captured by the medicine list builder	
Incorporating reminder functionalities such as: SMS reminders of due or overdue medications Periodic email reminders or mobile app notifications to users to update their medicines lists	
Allow provisions for user to register a community pharmacy in their online profile	

*Note*: SC members included: C1 and C2, the two older people; C3, carer; Ph1, Pharmacist; RN1, Nurse; G1, Geriatrician; GP1, General Practitioner; DHR1 and DHR2, Digital Health Researchers.

Abbreviations: HCPs, health‐care practitioners; SC, Stakeholder Committee; SMS, short message service.

### 3.3 Website usability across diverse populations

Developing a website tailored to the needs of older people, including those from diverse population groups, was the most recurrent issue. Members of the SC identified this as a priority for enhancing the user experience and promoting user engagement. A range of population groups were identified by the SC, including older people with polypharmacy, from culturally and linguistically diverse backgrounds, Aboriginal and Torres Strait Islander people, and older people with low digital literacy. Consumer SC members emphasised the need to involve carers and families in the co‐design of subsequent versions of the MVP.

Many old people have either a family member or carer that looks after, assists them with their medications … it is important that the study also captures the needs of carers … (C3)

### 3.4 Optimising the user experience

Eight of the nine SC members agreed that the suggested colour scheme and theme of the website was appropriate to the target audience and aesthetically pleasing. Whilst a majority of the SC members described the website look and feel as ‘great’, ‘clean’, having ‘soft colours’, ‘really like the big buttons and simple questions and options’, SC member, RN1, questioned if the proposed design was culturally sensitive towards Aboriginal and Torres Strait Islander peoples, and also noted that the SC did not include any Aboriginal and Torres Strait Islander representatives. To optimise the user experience, SC members suggested adopting large font sizes throughout the website, focussing on simplicity and use of consumer‐focussed language: ‘For consumer‐focussed language consider referring to users as a person rather than a patient’ (Ph1).

### 3.5 Priorities of SC consumer versus HCP members

Contributing their individual experiences and expertise, SC HCP members expressed concerns on the accuracy of consumers entering their medicines information via the online portal. For example, ‘it may be difficult for older people to manage their own medicines list with guaranteed accuracy – is the intended purpose of the list just for empowerment and information for the older person … ’ (Ph1). On the other hand, SC consumer members tended to emphasise the benefits of having an electronic medication list over a paper medication list, which ‘gets lost’ is ‘not always up to date’ and ‘may not be legible’. However, one of the consumer SC members (C2) introduced a new consideration regarding the limited role of MMG in emergency situations stating ‘a medicine list like this is not good for (an) emergency because no one else can see what the patient is taking’ due to email and password login restrictions.

In light of the above, the SC recommended that the remaining co‐design phases, including testing of subsequent versions of the website, should not only involve older people and informal carers, but also HCPs caring for older people. Engaging HCPs such as general practitioners and community pharmacists in the creation of online PDS tools may contribute to more effective tools being developed that promote enhanced collaboration and communication between older people and HCPs.

### 3.6 The Medicines List builder

The medicines list builder on the website was a major focus of SC MVP review meetings and email correspondence. Stakeholder committee member DHR2 echoed the significance of developing a user‐friendly online medicines list, drawing from their previous qualitative work yielding outcomes suggestive of older people preferring electronically capturing their medicines information. Key considerations identified by the SC for the development of an online medicines list builder included having an option for barcode scanning of medications and prescriptions to capture medicines information more accurately and integrating MMG online medicines list with existing platforms like My Health Record.^22^ There was division among SC members regarding preferences for listing medication names by active ingredient or brand names. Due to the vast number and dynamic nature of medication brands and the logistical difficulties this would pose from a web build perspective, a suggestion to develop an education video for users of the website to distinguish between active ingredient names and brand names was put forward by DHR1 and accepted by all members as the consensus option.

## 4 DISCUSSION

In this paper, we describe the first steps leading to the development of a preliminary version of a new online PDS for older people to manage their medications (MMG website) and discuss the involvement and engagement of a SC. Co‐design has become increasingly valued within health‐care research; albeit, there are few studies outlining how co‐design can be effectively applied, especially with older people, in the development of health technologies.^23^ Our work addresses this important knowledge gap by contributing an approach that enables older people to be active contributors as SC members. Collaborative approaches empowering end‐users to more actively contribute their unique experiences and knowledge are key to the success of new interventions.^24^ Such collaborative effort involving all stakeholders seems particularly important in the context of creating online health solutions for older people, because as the proportion of older internet users continues to rise, more and more technology‐savvy individuals are expected to enter the older‐age population.^25^

Moreover, consistent with the current literature on electronic health tools^26^ including online PDS tools, the high level of engagement observed from our SC consumer members supports the notion that older people and carers are willing to engage online PDS tools to enhance self‐management of their health and medications. Adapting online PDS tools to the needs of older users is essential to meeting their needs because cognitive decline, reduced locomotor ability and visual impairment may inhibit screen reading abilities, retention of information and use of a computer mouse or touch pads.

Significantly, our findings also confirm previous research^15^ that has identified differences in the priorities between patient and HCP narratives, emphasising the importance of engaging all stakeholders in co‐design. Our findings suggest that while HCPs tended to focus on the accuracy of medication information entered in the online PDS to support clinical decision‐making, older people focussed on the educational aspects and accessibility of the PDS. Drawing from the themes identified, presented below are enablers and barriers to effectively engaging older people in co‐design.

### 4.1 Enablers

According to Darley and Carroll,^27^ an enabler to facilitating genuine co‐design with users, including older people, is empowerment through relationship and trust‐building. As equal partners in the research work and to feel their contributions are valued, they are empowered to actively participate in shaping research outputs that are relevant and adapted to meeting their needs.^11^, ^23^ Recruiting older people and carers as members of the SC responsible for governing the research project was strategic to empower the older SC consumer members to view themselves as core contributors in the development of the PDS. Strategies employed in this research to foster relationship and trust‐building included having check‐ins at the beginning of meetings, dividing the SC into smaller groups (via breakout rooms) and acting on feedback.

Additionally, partnership with a diverse stakeholder group is a critical enabler for effective co‐design, recognising all members as experts of their own experiences whilst promoting mutual learning.^26^ Recruiting a range of expert groups for our SC, including older people, carers, individuals from culturally and linguistically diverse backgrounds and HCPs, promoted an environment for members to apply their lived experiences, tacit knowledge and ideas to develop a tool that meets their needs. Recruitment from geographically diverse groups was enabled by videoconferencing. Furthermore, the size of the SC, individual members' inherent biases, and perhaps what SC members may have considered assumed knowledge may have also contributed to the points of diversity that arose in SC meetings.

### 4.2 Barriers

Research suggests that co‐design with marginalised and vulnerable populations, including older people, may be challenging due to individuals from such population groups perceiving themselves as unqualified or unsuitable candidates for co‐design.^23^ Adopting a methodology like the one presented in this paper enables project investigators to collaborate with older people first on a smaller scale (as members of a SC) foreshadowing future phases of the co‐design work with larger groups of older people to develop subsequent versions of the desired interventions.

### 4.3 Comparison with previous work on online medication management PDS tools

Like other existing online tools identified in the published literature,^28^ this novel PDS MMG allows older people and carers to record medication information via the online medicines list builder, includes functionalities for patients to self‐report medication management information or changes, including symptoms and indications, health data and adverse drug events, and provides users access to a range of consumer‐friendly health and medication information. However, MMG goes a step further to support older people in developing individualised and SMART health and medication goals. To our knowledge, existing medication management PDS tools for older people do not incorporate a SMART medication‐related goals‐of‐care functionality although previous research^29^ on the development of health promotion interventions for older people has identified SMART goals as relevant. It is hypothesised that a platform such as MMG will empower older people to make informed decisions for their health and promote shared decision‐making with HCPs, thus contributing to improved health outcomes.

### 4.4 Study limitations and implications for future research

A limitation of this study is that despite the diverse SC composition, our SC may not be representative of all older people; therefore, our results may not be generalisable. Therefore, next steps for this project will include progressing through the remaining co‐design phases (2–4) to involve focus groups and/or interviews with larger groups of older people. Furthermore, the investigators did not utilise the traditional thematic analysis approach; rather, a brief content analysis was considered sufficient to identify common topics generated from SC meetings and feedback. Future work involving co‐design with larger groups of end‐users to develop subsequent high‐fidelity versions of MMG will benefit from more robust data analysis.

Additionally, further work examining the usability and utility of MMG in diverse populations is required to adequately address topics raised by our SC regarding website usability and optimising user experience. Similarly, future research to test the hypothesis that users engaging with the SMART goals‐of‐care functionality in MMG are associated with improved health outcomes is required.

## 5 CONCLUSIONS

Collaboration with the SC was crucial in developing a preliminary version of an online PDS for older people that will form the working draft for subsequent co‐design work. Key considerations for developing online PDS for older people that emerged from our partnership with a SC include enhancing website usability across diverse older‐age populations, incorporating appropriate website design, intuitive interfaces and consumer‐focussed language for the optimal user experience.

## CONFLICT OF INTEREST STATEMENT

No conflicts of interest declared.

## ETHICS STATEMENT

The Northern Sydney Local Health District Human Research Ethics Committee waived the need for ethics approval as this project involved consultations with a stakeholder committee, in an advisory capacity, without engaging in any activities that would necessitate ethical oversight.

## Supporting information


Data S1

## Data Availability

The data that support the findings of this study are available upon request from the corresponding author. These data are not publicly available due to privacy or ethical restrictions.
