# Supplementary material for: First steps in co‐designing an online patient decision support tool for enhanced medication management in older people
Source: Australas J Ageing. 2025 Jun 13;44(2):e70053. doi: 10.1111/ajag.70053 (PMC12163865; doi:10.1111/ajag.70053)
Supplement: Supplementary file 1 — Data S1 [file AJAG-44-0-s001.docx]

**Appendix 1:** Stakeholder committee meeting discussion questions.

**Stakeholder Committee meetings 1 and 2* Breakout group questions- October 2023**

**Q1. In your groups, discuss the below in relation to My Medicine Goals (MMG) concept**

**and design.**

• ways to enhance the user-experience (e.g. aesthetics, language, font, colours,

images)

• challenges older people may face with the current design and provide possible

solutions.

**Q2. Review MMG website content:**

• What would you change to ensure the website is fit for purpose?

• What do you anticipate would be most important for an older person using a

medicines website?

• What do you think an older person using a medicines risk calculator would like it

to do for them?

**Q3. Is there anything else you would like to raise?**

*****NB Stakeholder committee meetings 1 and 2 were held on 11/10/23 and 12/10/23 to

accommodate for SC member availabilities. The same questions were discussed at

both meetings.
